# Magnetic Assembly Route to Construct Reproducible and Recyclable SERS Substrate

**DOI:** 10.1186/s11671-019-3184-7

**Published:** 2019-12-05

**Authors:** Bingfang Zou, Chunyu Niu, Ming Ma, Lu Zhao, Yongqiang Wang

**Affiliations:** 1 Key Laboratory for Special Functional Materials of the Ministry of Education, Henan University, Kaifeng, People’s Republic of China; 2 School of Physics and Electronics, Henan University, Kaifeng, People’s Republic of China

**Keywords:** Magnetic, Assembly, Multifunctional microsphere, SERS, Recyclable

## Abstract

The fabrication of a uniform array film through assembly of colloidal building blocks is of practical interest for the integrated individual and collective functions. Here, a magnetic assembly route was put forward to organize monodisperse noble metal microspheres into a uniform array film for surface-enhanced Raman scattering (SERS) application, which demonstrated the integrated signal sensitivity of single noble metal microspheres and reproducibility of their assembled uniform array film. For this purpose, monodisperse multifunctional Fe_3_O_4_@SiO_2_@TiO_2_@Ag (FOSTA) colloidal microspheres as building blocks were successfully synthesized through a homemade ultrasonic-assisted reaction system. When used in SERS test, these multifunctional microspheres could firstly bind the analyte (R6G) from solution and then assembled into a uniform film under an external magnetic field, which exhibited high SERS detection sensitivity with good reproducibility. In addition, due to the TiO_2_ interlayer in FOSTA colloidal microspheres, the building blocks could be recycled and self cleaned through photocatalytic degradation of the adsorbed analyte for recycling SERS application.

## Introduction

Due to the unexampled advantages of the integration of unique spectroscopic fingerprint, high sensitivity, and nondestructive data acquisition, the surface-enhanced Raman scattering (SERS) spectroscopy has been intensely explored as a powerful and extremely sensitive analytical technique with wide potential applications in biochemistry, chemical synthesis, food safety, environmental monitoring, and so on [1–3]. Since it was first discovered that a rough silver metal surface could greatly enhance the Raman scattering spectroscopy of adsorbed molecules, the SERS substrate has always been the research focus for its strong relation with Raman signal [4, 5]. The gaps or junctions in the aggregates, called “hot spots” later, was found to contribute to strong Raman signals [6], and then great progress has been made in the design and synthesis of various noble metal nanostructured materials with “hot spots” containing structures [7].

Until now, various materials composed of SERS-active NPs and nanostructured support materials have been designed for higher enhancement [8, 9]. In general, SERS substrates can be classified into two major categories: structured films and colloidal particles. For structured films, they were often fabricated through complex procedures like electron beam lithography, AAO template, and colloidal array template like polystyrene [10–14], and the surface of these structured films were rather uniform which is beneficial for improved reproducible SERS signals. However, the fabrication procedure was time consuming, and it is also difficult to prepare nanopatterned surfaces with controllable nanogaps that are smaller than 5 nm [15]. Therefore, the SERS enhancement of structured surfaces is typically much less than that of noble metal particles or hierarchical microspheres prepared by wet chemistry methods since dense nanoscaled gaps bestrewed the whole hierarchical particles [16–18]. Unfortunately, although the signal sensitivity of hierarchical noble metal particles as SERS substrate was excellent, their reproducibility was relatively poor due to disorder aggregation [19].

To solve the irregular “hot spot” distribution, the self-assembly strategy is utilized to realize ordered aggregation, which induced the generation of relatively uniformly distributed “hot spots” between nanoscaled building blocks [20, 21]. Various methods are developed based on different forces including surface tension, covalent interactions, and Van der Waals and electrostatic attraction forces [22–29]. For examples, Bai et al fabricated large-area arrays of vertically aligned gold nanorods through a controlled evaporation deposition process [23]. Kim et al reported a simple method to fabricate an ultrahigh-density array of silver nanoclusters as SERS substrate with high sensitivity and excellent reproducibility based on PS-b-P4VP micelles [28]. These reported film assembly of the noble metal particles demonstrate highly reproducible SERS signals, but the binding rate of analytes is lower in comparison with the suspension approach.

Magnetic noble metal microspheres could capture analyte efficiently in solution through magnetic separation and exhibited excellent SERS performance after they were immobilized on a glass slide [30–32]. Furthermore, photocatalytic materials were also introduced to create self-cleaning SERS substrate, which makes the SERS substrates easily recyclable [33, 34]. Unfortunately, although these multifunctional magnetic composite microspheres could bind analyte and form films quickly under an external magnetic field, the resultant film was often in disorder, which resulted in a very uneven distribution of “hot spots” and poor SERS signal reproducibility. Thus, all these magnetic noble metal microspheres in SERS application are just limited to act as a magnetic separation tool. Although magnetic assembly was attractive for its simple manipulation under external magnet, it requires high monodispersity of the building blocks, especially for three-dimensional assembly [35]. Until now, no study has reported using the magnetic assembly route to construct reproducible and recyclable SERS substrate.

**Scheme 1:**
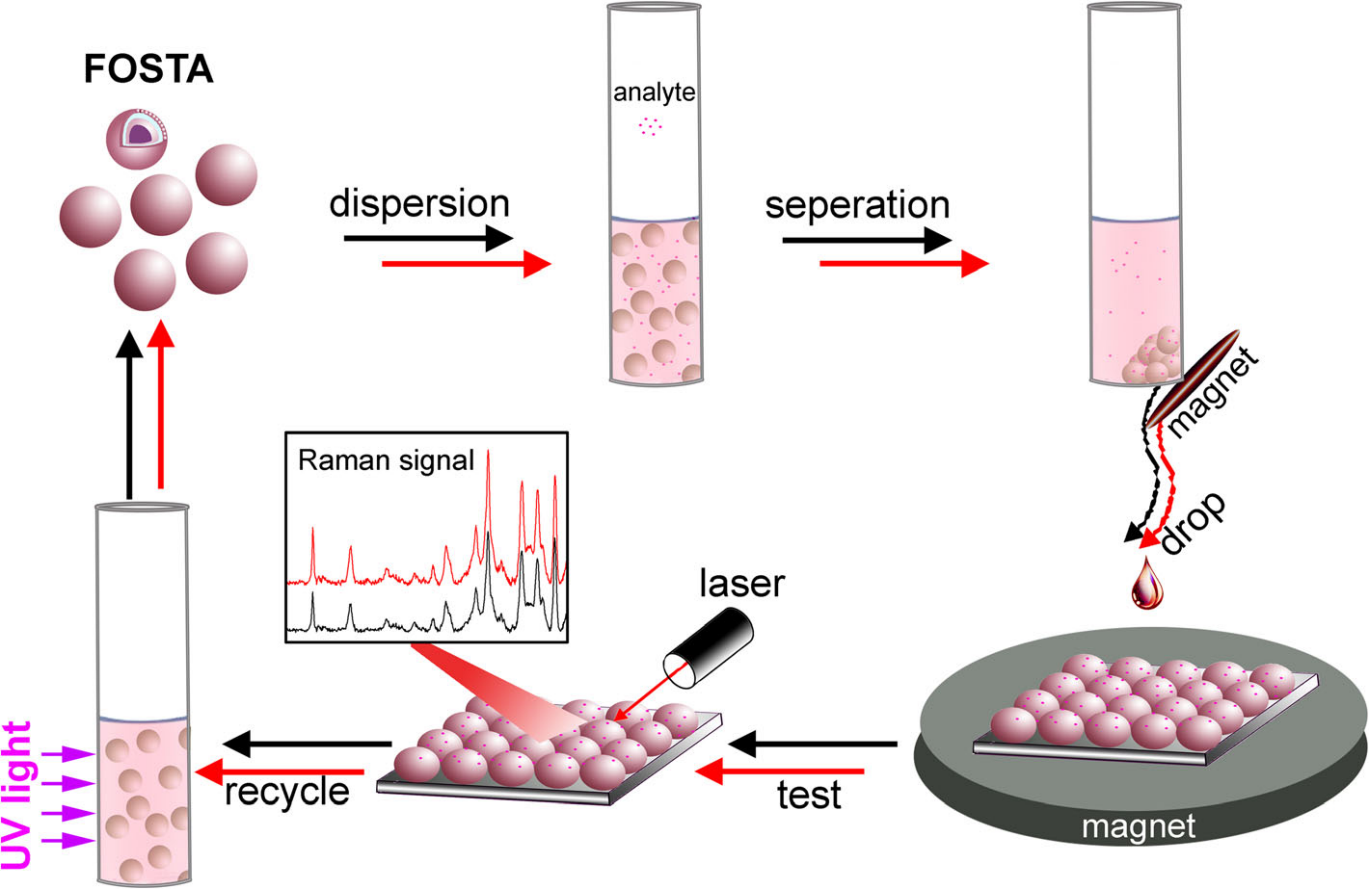
Magnetic manipulation of multifunctional FOSTA composite microspheres for reproducible and recyclable SERS substrate

Herein, monodisperse multifunctional Fe_3_O_4_@SiO_2_@TiO_2_@Ag (FOSTA) composite microspheres were successfully synthesized in a homemade ultrasonic-assisted reaction system, which are suitable building blocks for magnetic assembly. As Scheme 1 demonstrates, the multifunctional FOSTA composite microspheres could efficiently capture the analyte (R6G) from the solution through dispersion and magnetic separation firstly for SERS analysis. And then, these FOSTA composite microspheres were assembled into a uniform film on a glass slide with external magnetic field, which is expected to exhibit highly sensitive and reproducible SERS performance. Furthermore, the used FOSTA composite microspheres can be recycled through photocatalytic degradation of the adsorbed analyte under UV irradiation.

## Experimental Section

### Synthesis of Fe_3_O_4_@SiO_2_@TiO_2_ Microspheres

Monodisperse Fe_3_O_4_@SiO_2_ microspheres were synthesized through our previous report [36]. The TiO_2_ shell coating was conducted in an ultrasonic tank to avoid aggregation. In a typical synthesis, Fe_3_O_4_@SiO_2_ (20 mg) was dispersed in a mixture of hydroxypropyl cellulose (0.1 g), ethanol (45 mL), and deionized water (0.1 mL). After 30 min, 1 mL of tetrabutoxy titanium in ethanol (5 mL) was completely injected into the mixture using a peristaltic pump for 15 min. And then, the water in the ultrasonic tank was heated to 85 °C gradually and refluxed for 100 min. After the product was separated by an external magnet and washed repeatedly in ethanol, it was redispersed into 75 mL deionized water containing polyvinylpyrrolidone (PVP, 1.0 g) under ultrasonic for 30 min, and then, the solution was transferred into a Teflon autoclave to transform an amorphous TiO_2_ shell into an anatase structure at 180 °C.

### Synthesis of FOSTA Microspheres

The silver shell coating was also conducted in an ultrasonic tank to avoid aggregation. The above Fe_3_O_4_@SiO_2_@TiO_2_ microspheres (about 25 mg) were dispersed in a water/ammonia/ethanol mixed solution (2 mL/0.2 mL/13 mL) containing AgNO_3_ (0.1 g) and PVP (1 g), and then, the whole solution was dispersed with the aid of an ultrasound for 30 min at 40 °C. The temperature was then increased to 85 °C. The flask bottle was taken out from the ultrasonic tank after a certain time, and the product was instantly separated by an external magnet and then washed in ethanol for several times. The final product was saved in ethanol for further characterization and usage.

### Characterization

The products were analyzed by X-ray diffraction (XRD), in a 2θ range from 10° to 80°, using Cu Kα radiation (Philips X’pert Diffractometer), scanning electron microscopy (SEM, Hitachi S-4800), and transmission electron microscopy (TEM, JEOL-2010). Magnetic measurements were performed with a superconducting quantum interference device magnetometer (SQUID, Quantum Design, MPMS XL).

### SERS Measurements

R6G was used as Raman probe to test the reproducibility of the SERS substrate. The R6G solution (20 mL) with different concentrations were firstly prepared, and the above as-prepared FOSTA composite microspheres stocked in ethanol were added and placed on the shaking bed for 2 h. and then, the product was extracted by an external magnet and washed in ethanol. The remaining solution was dropped on a cleaned silicon pellet with a circular magnet under it, and then, the solution was covered by a Petri dish and left alone until all ethanol evaporated. The whole process was conducted on an antishock platform. After the residual solution was slowly dried in air, the substrates were measured under the Raman instrument (LABRAM-HR), with its laser at an excitation wavelength of 633 nm in this study. The laser spot focused on the sample surface was about 3 μm in diameter, and the acquisition time was 3 s for each spectrum.

### Photocatalytic and Recycling Test

The photocatalytic performance of the as-obtained FOSTA composite microspheres was tested using R6G as model. The samples (40 mg) were dispersed in the R6G solution (40 ml, 10^-5^ M) and kept in the dark for 30 min for dark adsorption experiment. And the above solution was divided into eight equal aliquots, and put in the homemade photocatalytic setup with a 300-W mercury lamp as light source. One aliquot (5.0 mL) at respective irradiation time intervals was collected and centrifuged to remove the photocatalyst. The supernatant was analyzed quantitatively by measuring the absorbance at 525 nm on an ultraviolet–visible absorption spectrometer (Shanghai Instrument Analysis Instrument Co., Ltd.). The recycling tests are performed according to the above procedure except that UV exposure time was set for 100 min, and the sample was rinsed with deionized water several times to remove residual ions before SERS test.

## Results and Discussion

**Fig. 1:**
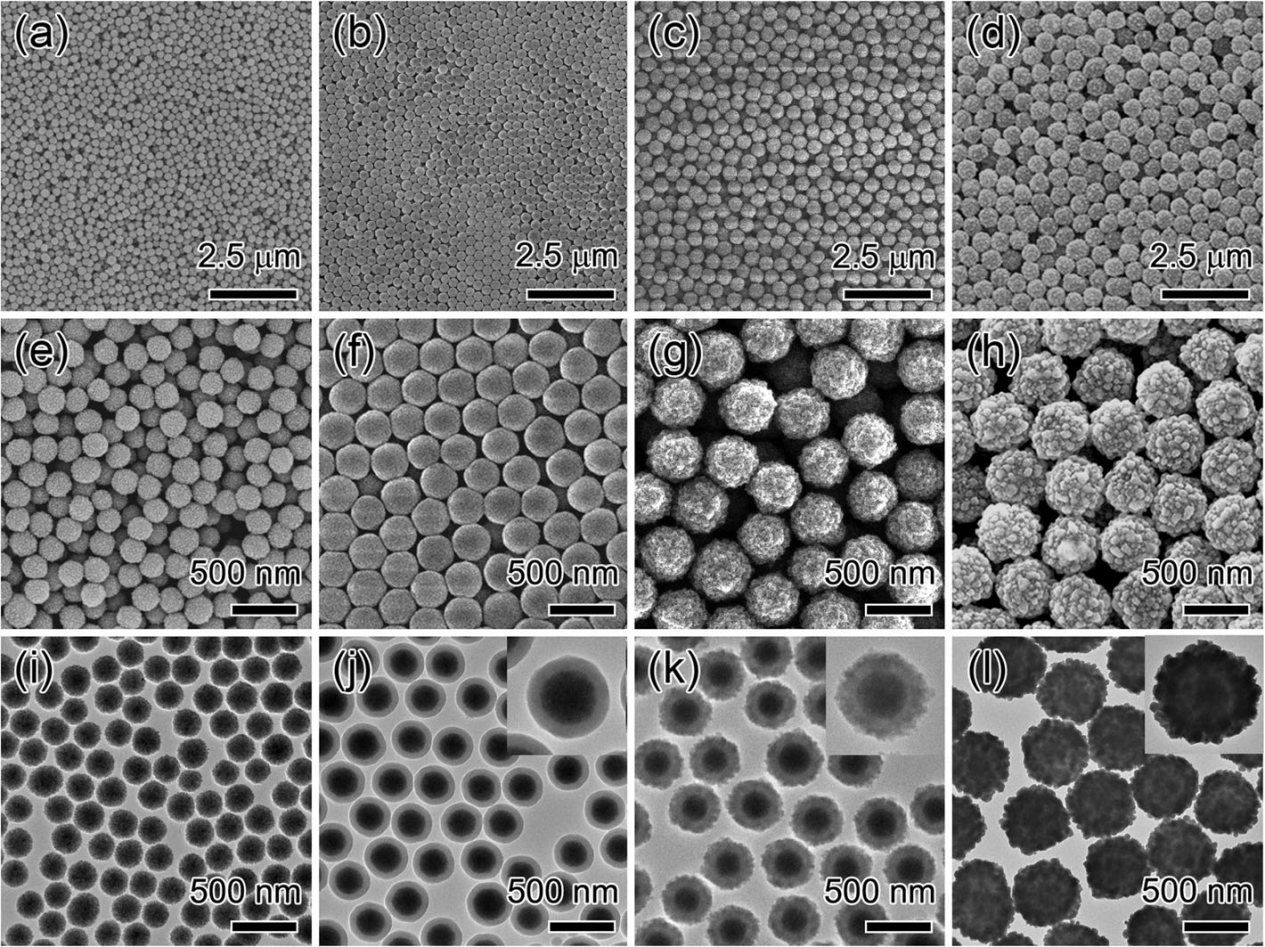
The SEM and TEM images of (**a**, **e**, **i**) Fe_3_O_4_, (**b**, **f**, **j**) Fe_3_O_4_@SiO_2_, (**c**, **g**, **k**) Fe_3_O_4_@SiO_2_@TiO_2_, and (**d**, **h**, **l**) Fe_3_O_4_@SiO_2_@TiO_2_@Ag microspheres, respectively

According to our designed route, monodisperse Fe_3_O_4_ microspheres are critical factors for magnetic assembly. Here, they were synthesized through a hydrothermal method as reported by our group previously [36]. As shown in Fig. 1a, e and j, monodisperse Fe_3_O_4_ microspheres with a diameter of 200 nm were synthesized, and they were dispersed very well without obvious aggregation. To further improve the particles' dispersion and compatibility for outer shell growth, the silica layer was coated on Fe_3_O_4_ microspheres through the Stöber method. As shown from Fig. 1b, uniform Fe_3_O_4_@SiO_2_ composite microspheres were obtained, and they tend to form hexagonally packed superstructures during the preparation of the SEM samples as a result of their high shape and size monodispersity as seen in Fig. 1b and f. Although uniform Fe_3_O_4_@SiO_2_ composite microspheres are a good candidate as magnetic platform for following shell growth, a special reaction system should be set up to avoid aggregation during TiO_2_ and Ag heterogeneous deposition, where mechanical stirrer and reflux were integrated in the ultrasonic tank in our experiment. The Fe_3_O_4_@SiO_2_@TiO_2_ composite microspheres synthesized without using mechanical stirrer and ultrasonic were showed in Additional file 1: Figures S1 and S2 in Supporting information, and aggregated particles or particles with a rough surface were observed due to the decreasing repulsive force between particles or inhomogeneous reaction solution during shell coating [37]. And well-dispersible Fe_3_O_4_@SiO_2_@TiO_2_ microspheres could be successfully fabricated in the homemade reaction system with both ultrasonic and mechanical stirrer as shown in Fig. 1c, and the shell was composed of tiny TiO_2_ nanoparticles in Fig. 1g and k. After the amorphous TiO_2_ shell was transformed into an anatase structure by hydrothermal treatment, they were further coated with silver through in-situ method, where silver ions were slowly reduced by PVP. The as-obtained FOSTA composite microspheres still kept well dispersed (Fig. 1d) and dense Ag nanoparticles were deposited on the TiO_2_ shell in Fig. 1h and i. From the above results, multifunctional FOSTA composite microspheres were synthesized through a multi-step coating procedure using the homemade setup. The mechanical stirrer together with reflux ensured that the reaction proceeds homogeneously, wherein, ultrasonic guaranteed the magnetic core to be well dispersed during the coating process. In summary, monodisperse multifunctional FOSTA composite microspheres were synthesized which could be used as building blocks for magnetic assembly.

**Fig. 2:**
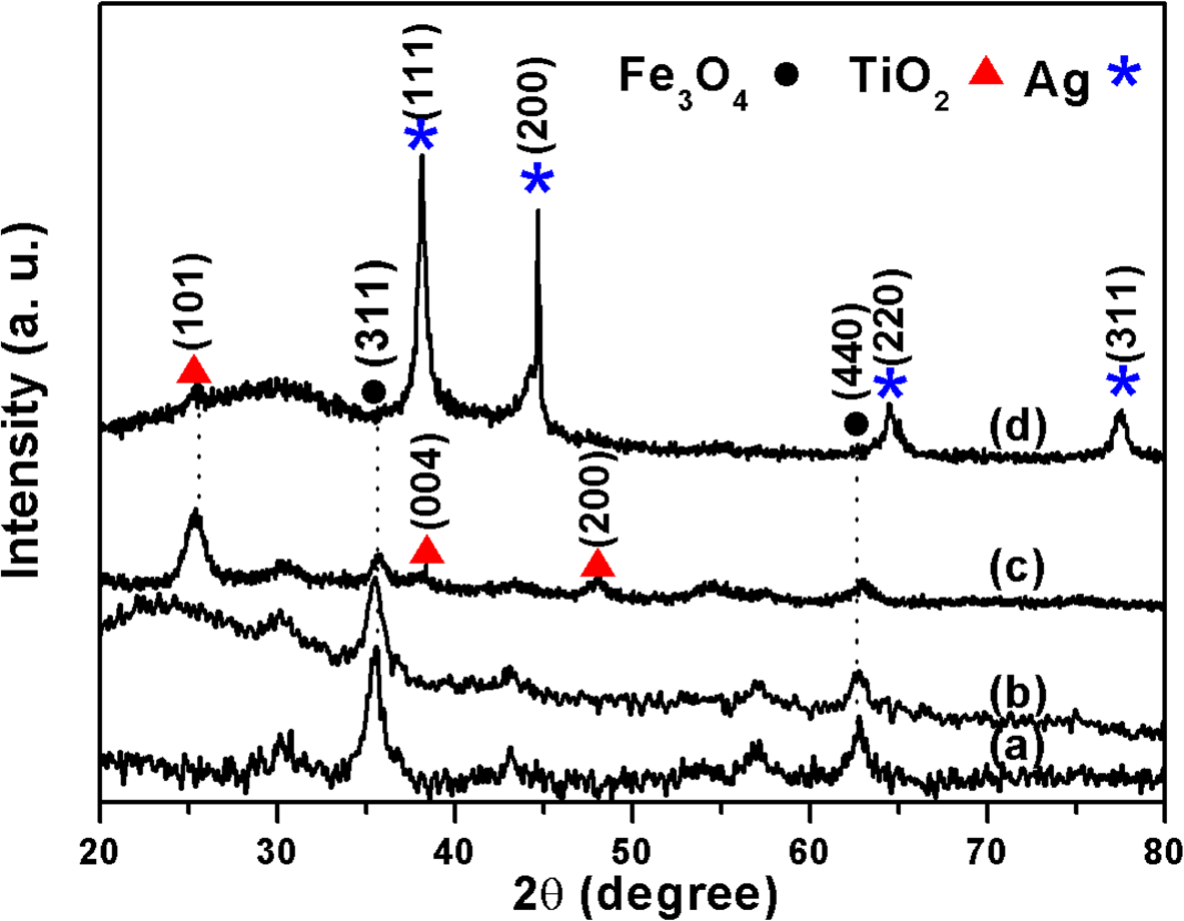
The XRD patterns of (**a**) Fe_3_O_4_, (**b**) Fe_3_O_4_@SiO_2_, (**c**) Fe_3_O_4_@SiO_2_@TiO_2_, and (**d**) Fe_3_O_4_@SiO_2_@TiO_2_@Ag, respectively

The above as-synthesized products at each coating step were all characterized by X-ray powder diffractometer (XRD). The specific XRD of Fe_3_O_4_ in Fig. 2a is characterized by two peaks positioned at 35.3° and 62.4° (black dots), which correspond to the (311) and (440) lattice planes of the cubic phase of Fe_3_O_4_ (JCPDS Card no. 75-0449), respectively. After being coated with amorphous SiO_2_ layer, a wide peak centered at 23° was observed in Fig. 2b [38]. When another layer of TiO_2_ was deposited and treated hydrothermally, the XRD pattern of the Fe_3_O_4_@SiO_2_@TiO_2_ microspheres showed several additional peaks located at 25.3°, 37.9°, and 48.0° (red triangles) in Fig. 2c compared with that of Fe_3_O_4_@SiO_2_ microspheres, which corresponded to the reflections from the (101), (004), and (200) planes of the anatase phase (JCPDS card no. 75-2545). After depositing dense Ag nanoparticles on the surface of the Fe_3_O_4_@SiO_2_@TiO_2_ microspheres, the diffraction peaks of the above materials could still be observed but faintly due to the strong peaks at 38.1° and 44.6° (blue stars) in Fig. 2d, which were indexed as (111) and (200) of the cubic phase of Ag (JCPDS card, no. 4-783). The XRD patterns in Fig. 2 show that the characteristic diffraction peaks correspond to the spinel Fe_3_O_4_, amorphous SiO_2_, anatase TiO_2_. and cubic phase Ag NPs in the FOSTA composite microspheres. The characteristic XRD patterns indicated that three different layers were successively coated on Fe_3_O_4_ microspheres which were consistent with the designed route.

**Fig. 3:**
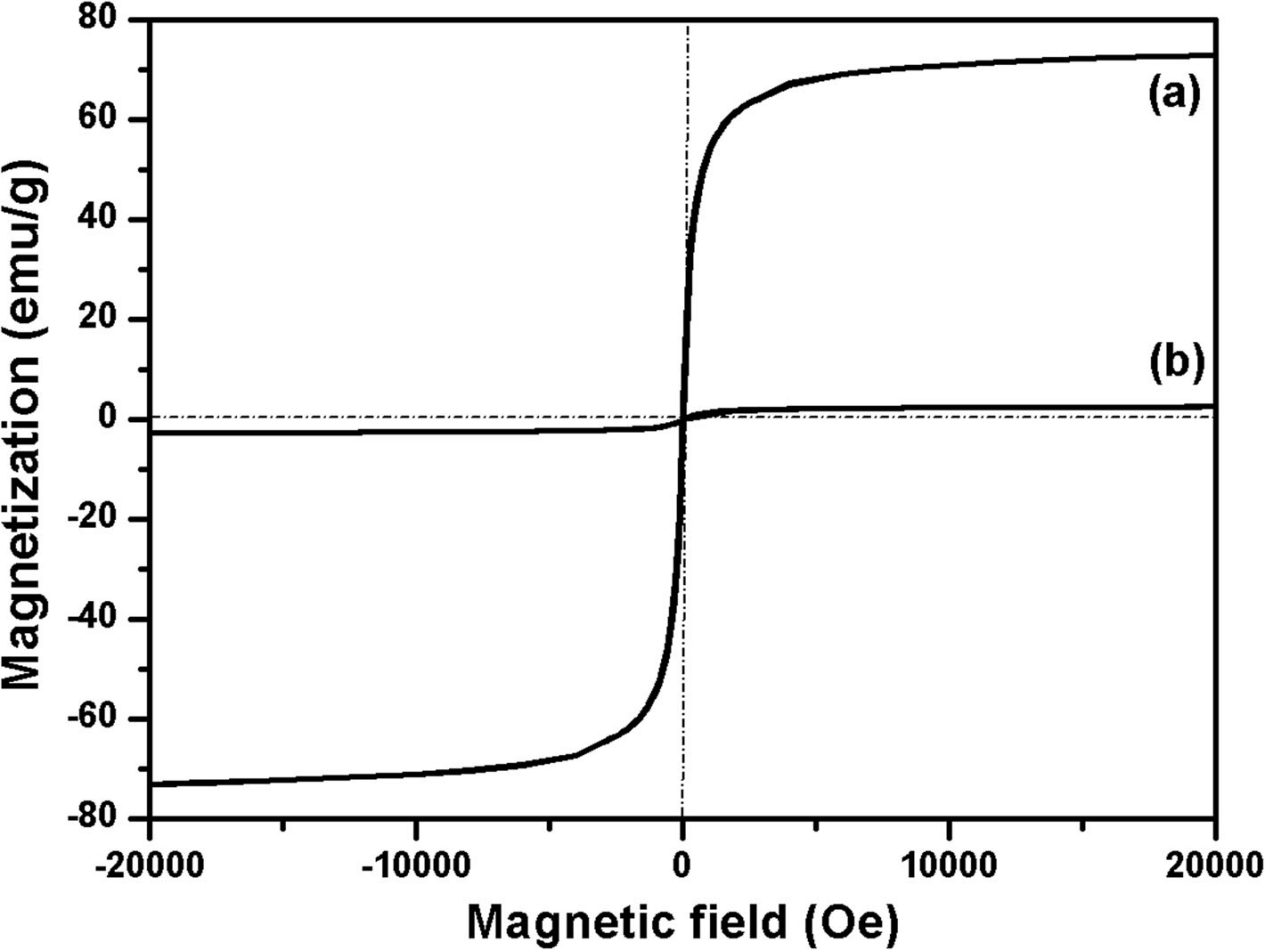
Room temperature magnetic hysteresis curves of (**a**) Fe_3_O_4_, and (**b**) FOSTA composite microspheres

The magnetic properties of the Fe_3_O_4_ and FOSTA composite microspheres were investigated, as shown in Fig. 3. The zero coercivity and the reversible hysteresis behaviors, shown in Fig. 3a, indicated the superparamagnetic nature of the Fe_3_O_4_ microspheres. The room-temperature saturated magnetization of Fe_3_O_4_ microspheres are 73.3 emu/g, but the magnetization of FOSTA composite microspheres, which were inherited from the magnetic Fe_3_O_4_ particles, decreased obviously due to the extra nonmagnetic materials, including SiO_2_, TiO_2_, and Ag shells. Although their saturated magnetization value (2.62 emu/g) decreased greatly, the FOSTA composite microspheres still could be packed from a suspension system slowly by magnetic separation.

**Fig. 4:**
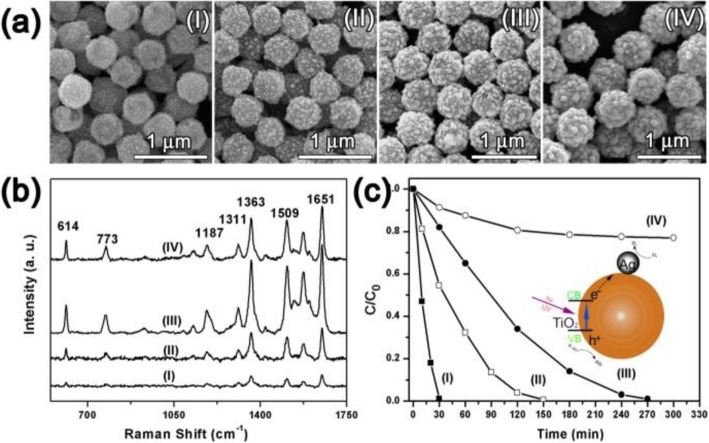
The SEM images (**a**), SERS spectra (**b**), and photocatalytic performance (**c**) of FOSTA composite microspheres at different reaction times (I) 15, (II) 20, (III) 25, and (IV) 30 min

In the FOSTA composite microspheres, the Ag shell structure was important because it not only determined the SERS performance, but also influenced the photocatalytic properties, thus, the controlled growth of the Ag shell was necessary for optimizing the overall performance. Here, PVP acted as a kind of mild reducing agent except surfactant, thus the growth of Ag nanoparticles could be easily controlled by the reaction time after the Ag nuclei appeared on the Fe_3_O_4_@SiO_2_@TiO_2_ microspheres. Four typical products at different intervals were sampled and investigated, which were named as samples I–IV (Fig. 4). As shown in Fig. 4a, tiny Ag nanoparticles appeared at 15 min, and then, these Ag nanoparticles grew large with reaction proceeding 20 min, but they did not contact each other. With the continual growing up of Ag nanoparticles, most of the surface of Fe_3_O_4_@SiO_2_@TiO_2_ microspheres was covered at 25 min. Finally, the surface of Fe_3_O_4_@SiO_2_@TiO_2_ microspheres were fully covered by large Ag nanoparticles. During the growth procedure, it can be seen that the Ag nanoparticles on the surface of Fe_3_O_4_@SiO_2_@TiO_2_ microspheres grew gradually from Ag nanoparticles to a complete shell.

The above samples at different intervals were firstly tested as SERS substrate, using R6G as probe, and the corresponding results are shown in Fig. 4b. All the peaks from 500 to 1750 cm^-1^ in Fig. 4b were indexed as R6G signals, where the peak at 773 cm^-1^ is due to the out-of-plane bending motion of the hydrogen atoms of the xanthenes skeleton, and other peaks at 1187, 1311, 1363, 1509, and 1651 cm^-1^ are assigned to C–H in-plane bending, C–O–C stretching, and C–C stretching of the aromatic ring [39]. The strongest peak at 1363 cm^-1^ was chosen for comparison. Sample I showed a very weak SERS signal with no clear distinguishable peaks since these separated Ag nanoparticles were too small. Sample II exhibited a stronger SERS signal than that of sample I for the local EM enhancement increases with increasing particle size [40, 41]. The SERS signal of sample III was further enhanced because the size of these separated Ag nanoparticles reached about 50 nm, which was reported to produce the strongest enhancement [40]. Besides, these Ag nanoparticles get close together which created a large amount of gap as “hot spots” [8]. However, the continual growth of Ag nanoparticles finally made them merge together in sample IV, and the gaps disappeared at the same time, which then decreases their SERS activity. Therefore, sample III shows the highest SERS performance when compared with other samples.

The photocatalytic performance of samples I–IV were then investigated using R6G. As seen in Fig. 4c, R6G could be degraded totally by samples I–III under UV irradiation. As shown in the inset of Fig. 4c, the TiO_2_ core of TiO_2_–Ag composites on magnetic microspheres can be excited under UV light, and the electrons were transferred from the TiO_2_ conduction band to the Ag conduction band and then generate highly active oxidative species, such as •O_2_^-^ and •OH. These oxidative species can subsequently lead to the degradation of R6G [42]. However, with increasing Ag content in the FOSTA composite microspheres, the degradation capability of samples I–IV demonstrated a decreasing tendency. Previous studies show that the noble metal nanoparticles loaded on TiO_2_ with an optimized size and density are necessary to achieve excellent catalytic performance. And larger Ag content could be detrimental to the photodegradation performance since the Ag particles can also act as recombination centers. Thus, the total degradation time became longer with the increasing Ag content in our experiments, and it was up to almost 3 h for sample III. Although sample III shows a relative weaker degradation efficiency, the absorbed R6G molecules could still be completely degraded which met the requirement of self cleaning. Therefore, sample III with the strongest Raman enhancement should be the optimal building block for assembled SERS substrate based on comprehensive consideration.

**Fig. 5:**
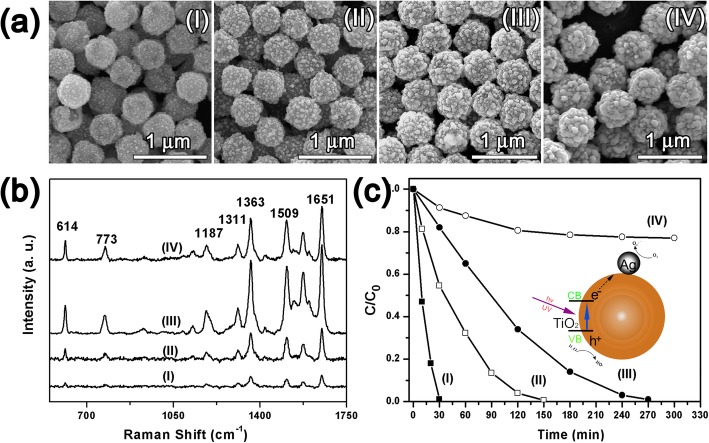
The SEM images of (**a**, **b**) typical magnetic-assembled film and (**c**, **d**) typical self-assembled film and their SERS signals reproducibility (**e**) and (**f**), respectively. The intensity distribution of peak at 1363 cm^-1^ from magnetic-assembled film and self-assembled film (**g**) and concentration-dependent SERS spectra of magnetic-assembled film (**h**)

Magnetic assembly was reported to be a very powerful assembly method since the magnetic packing force driven by the field gradient was able to induce the local concentration of particles and thus initialize the crystallization process [43]. Here, guided by the external magnetic field, the FOSTA composite microspheres (sample III, the same below) were assembled into ordered structures quickly and efficiently due to their superparamagnetic and monodisperse characteristics. As seen in Fig. 5a, the as-obtained monodisperse FOSTA composite microspheres could be successfully assembled into a large-area and uniform film under external magnet (named “magnetic-assembled film”), and hexagonal-packed structures could be observed from a magnified local area in Fig. 5b. For comparison, the film composed of FOSTA composite microspheres without external magnetic field (named “self-assembled film”) was also constructed, but a rough film with disordered structure was obtained in Fig. 5c, which was attributed to random aggregation during solvent evaporation in Fig. 5d. Besides, the magnetic-assembled film is smoother than the self-assembled film. The above results demonstrated a more uniform film including orderliness and smoothness, which could be obtained by magnetic assembly of the FOSTA composite microspheres. The reproducibility of SERS signals from films assembled with or without external magnetic field was investigated by choosing 20 spots across the substrate, as shown in Fig. 5e and f. The concentration of R6G solution was 10^-8^ M, and the corresponding Raman intensity (1363 cm^-1^) was recorded in Fig. 5g. The average relative standard deviation (RSD) of the magnetic-assembled film was calculated to be about 0.05, which was much lower than that of the self-assembled film with the value of about 0.197. It was also observed that the intensity of Raman peaks from the magnetic-assembled film are slightly higher than that from the self-assembled film on average, which could be attributed to secondary “hot spots” generated between particles, revealing an array structure-enhanced effect [44]. In summary, the above experimental results indicated that magnetic assembly endowed the FOSTA composite microspheres more advantages in both sensitivity and reproducibility. The concentration-dependent SERS spectra of R6G were further tested to investigate the detection limit of the FOSTA composite microspheres. The detection capabilities of the magnetic-assembled film were evaluated with R6G solutions over a wide range of concentration from 10^-6^ to 10^-12^ M. In Fig. 5h, the FOSTA composite microspheres exhibit obvious enhancement signals with concentrations from 10^-6^ to 10^-11^ M, and all enhancement peaks could be observed clearly even at a concentration as low as 10^-11^ M in the inset of Fig. 5c. The logarithmic intensity measured at 1363 cm^-1^ peak was plotted versus the logarithmic concentration of R6G (Additional file 1: Figure S3). The linear range for R6G detection was from 10^-6^ to 10^-11^ M with a limit of detection (LOD) of 10 ppb, revealing highly sensitive detection capability of the designed SERS system [45, 46].

**Fig. 6:**
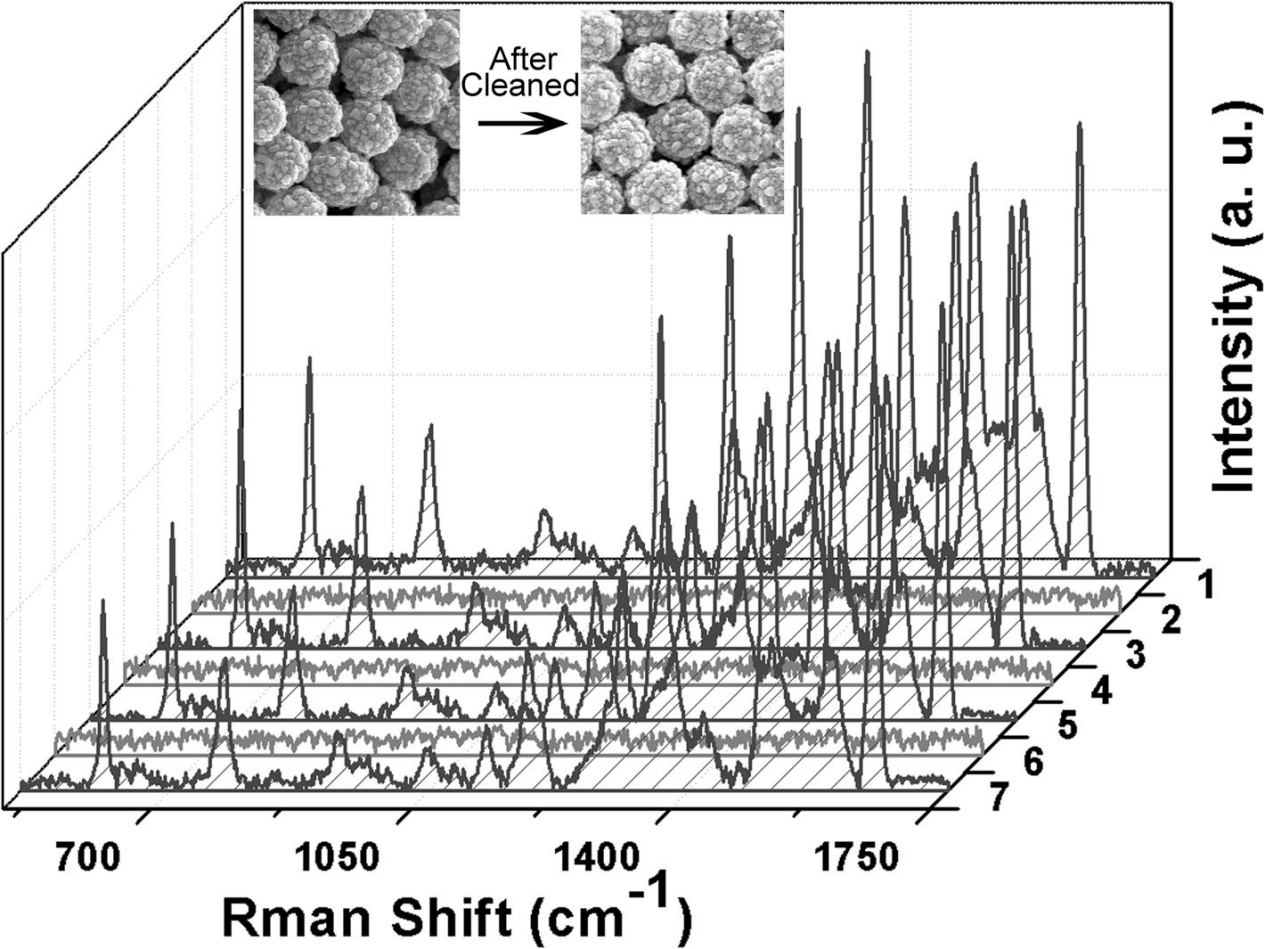
The recyclability of SERS substrate assembled by FOSTA composite microspheres

In our experiment, the SERS technology and photocatalytic properties were integrated through combining different functional layers including Ag and TiO_2_ shells. Their recyclability was studied by repeated SERS and photodegradation tests, as shown in Fig. 6. The FOSTA composite microspheres was first immersed in the solution containing the R6G analyte, then tested by SERS, and finally dispersed in deionized water with UV light for about 100 min. Then the sample was washed with deionized water several times to remove residual ions and molecules. It was observed that the main peaks disappeared, and the Raman spectra of the SERS substrate were similar to that of a new one. Obviously, it is very simple and easy to realize the self-cleaning goal since the amount of analyte absorbed onto the substrate is very low. After the substrate becomes clean, it can be used repeatedly several times. The SERS signals decreased little after three cycles from the Raman peaks of R6G, and no SERS signals were detected each time after self cleaning, which revealed that the FOSTA composite microspheres could be used as SERS substrate repeatedly. Furthermore, after three entire cycles, the morphology of FOSTA composite microspheres show no apparent change in morphology as seen from the inset images in Fig. 6, which implied that FOSTA composite microspheres were stable in physical strength.

## Conclusion

To create highly reproducible and recyclable SERS substrate, multifunctional microspheres were developed in the homemade ultrasonic-assisted reaction system as building blocks. Under the external magnetic field, the as-obtained FOSTA composite microspheres were assembled into a smooth and array-structured film, which exhibited sensitive and reproducible SERS performance. Due to the TiO_2_ shell, these used FOSTA composite microspheres could be further recycled through a self-cleaned procedure. Through integrating SERS and photocatalytic functions on magnetic microspheres, the magnetic assembly route is a promising technique for reproducible and recyclable SERS substrates.

## Supplementary information

**Additional file 1: Figure S1.** The Fe3O4@SiO2@TiO2 composite microspheres synthesized without using ultrasonic. **Figure S2.** A calibration curve where the logarithmic intensity measured at 1363 cm^-1^ peak is plotted versus the logarithmic concentration of R6G. Error bars are standard deviations calculated from 20 independent measurements. **Figure S3.** A calibration curve where the logarithmic intensity measured at 1363 cm^−1^ peak is plotted versus the logarithmic concentration of R6G. Error bars are standard deviations calculated from 20 independent measurements.

## Data Availability

Please contact author for data requests.
