# Supplementary material for: Magnetic Assembly Route to Construct Reproducible and Recyclable SERS Substrate
Source: Nanoscale Res Lett. 2019 Dec 5;14:369. doi: 10.1186/s11671-019-3184-7 (PMC6895331; doi:10.1186/s11671-019-3184-7)
Supplement: Supplementary file 1 — Additional file 1: Figure S1. The Fe3O4@SiO2@TiO2 composite microspheres synthesized without using ultrasonic. Figure S2. A calibration curve where the logarithmic intensity measured at 1363 cm-1 peak is plotted versus the logarithmic concentration of R6G. Error bars are standard deviations calculated from 20 independent measurements. Figure S3. A calibration curve where the logarithmic intensity measured at 1363 cm−1 peak is plotted versus the logarithmic concentration of R6G. Error bars are standard deviations calculated from 20 independent measurements. [file 11671_2019_3184_MOESM1_ESM.docx]

**Magnetic Assembly Route to Construct Reproducible and Recyclable SERS Substrate**

*Bingfang Zou*^a,b^*, Chunyu Niu*^a^*, Ming Ma*^a^*, Lu Zhao*^a^*, Yongqiang Wang*^a∗^

^a^ Key Laboratory for Special Functional Materials of the Ministry of Education, Henan University, Kaifeng 475004, P. R. China

^b^ School of Physics and Electronics, Henan University, Kaifeng 475004, P. R. China.

^∗^Corresponding author. Email: wangyq@henu.edu.cn

**

**

**Figure S1.** The Fe_3_O_4_@SiO_2_@TiO_2_ composite microspheres synthesized without using ultrasonic.





**Figure S2.** The Fe_3_O_4_@SiO_2_@TiO_2_ composite microspheres synthesized without using mechanical stirrer.

**

**

**Figure S3.** A calibration curve where the logarithmic intensity measured at 1363 cm^−1^ peak is plotted versus the logarithmic concentration of R6G. Error bars are standard deviations calculated from 20 independent measurements.
